# Insulin Sensitizing Pharmacology of Thiazolidinediones Correlates with Mitochondrial Gene Expression rather than Activation of PPARγ

**Published:** 2007-09-17

**Authors:** Charles W. Bolten, Patrick M. Blanner, William G. McDonald, Nicholas R. Staten, Richard A. Mazzarella, Graciela B. Arhancet, Martin F. Meier, David J. Weiss, Patrick M. Sullivan, Alexander E. Hromockyj, Rolf F. Kletzien, Jerry R. Colca

**Affiliations:** Discovery Research, Pfizer Corporation 700 Chesterfield Parkway West Chesterfield, MO 63017; 1 Kalamazoo Metabolic Research, 125 S. Kalamazoo Mall #604, Kalamazoo, MI 49007

**Keywords:** thiazolidinedione, insulin sensitizer, mechanism of action, mitochondria, diabetes, mitoNEET

## Abstract

Insulin sensitizing thiazolidinediones (TZDs) are generally considered to work as agonists for the nuclear receptor peroxisome proliferative activated receptor-gamma (PPARγ). However, TZDs also have acute, non-genomic metabolic effects and it is unclear which actions are responsible for the beneficial pharmacology of these compounds. We have taken advantage of an analog, based on the metabolism of pioglitazone, which has much reduced ability to activate PPARγ. This analog (PNU-91325) was compared to rosiglitazone, the most potent PPARγ activator approved for human use, in a variety of studies both in vitro and in vivo. The data demonstrate that PNU-91325 is indeed much less effective than rosiglitazone at activating PPARγ both in vitro and in vivo. In contrast, both compounds bound similarly to a mitochondrial binding site and acutely activated PI-3 kinase-directed phosphorylation of AKT, an action that was not affected by elimination of PPARγ activation. The two compounds were then compared in vivo in both normal C57 mice and diabetic KKAy mice to determine whether their pharmacology correlated with biomarkers of PPARγ activation or with the expression of other gene transcripts. As expected from previous studies, both compounds improved insulin sensitivity in the diabetic mice, and this occurred in spite of the fact that there was little increase in expression of the classic PPARγ target biomarker adipocyte binding protein-2 (aP2) with PNU-91325 under these conditions. An examination of transcriptional profiling of key target tissues from mice treated for one week with both compounds demonstrated that the relative pharmacology of the two thiazolidinediones correlated best with an increased expression of an array of mitochondrial proteins and with expression of PPARγ coactivator 1-alpha (PGC1α), the master regulator of mitochondrial biogenesis. Thus, important pharmacology of the insulin sensitizing TZDs may involve acute actions, perhaps on the mitochondria, that are independent of direct activation of the nuclear receptor PPARγ. These findings suggest a potential alternative route to the discovery of novel insulin sensitizing drugs.

## Introduction

Thiazolidinedione insulin sensitizers originated with an empirical discovery by the Takeda Company in the late 1970s. Modified analogs of lipid lowering drugs were found to lower both glucose and insulin levels in rodent models of type 2 diabetes (Sohda et al. 1984, 1990, 2002) secondary to improved insulin sensitivity (Hofmann and Colca, 1992; Owens, 2002). The first group of thiazolidinedione compounds that were eventually developed into clinical candidates was chosen solely using structure-activity relationships of pharmacology relative to safety in vivo. These compounds included troglitazone, which was subsequently removed from the market because of a unique idiosyncratic hepatotoxicity, and two compounds, rosiglitazone and pioglitazone, which are routinely used to treat type 2 diabetes (Day, 1999; Gillies and Dunn, 2000). These compounds are generally assumed to work as direct activators of the transcription factor peroxisome proliferative activated receptor-gamma (PPARγ) (Olefsky and Saltiel, 2000; Wilson et al. 1996) and new candidates have been both selected and evaluated based on their ability to directly activate or partially activate gene transcription directed by PPARγ (Leff and Reed, 2002; Sorbera et al. 2001). However recent evidence suggests that at least some of the pharmacology of the insulin sensitizers may be independent of PPARγ (Crosby et al. 2005, Feinstein et al. 2005, Konrad et al. 2005). Furthermore, Brunmeier and colleagues have shown that a number of these compounds (thiazolidinediones and non-thiazolidinedione analogs) have mitochondrial effects regardless of whether they are PPAR agonists or antagonists (Brunmair et al. 2001, 2004). Similarly it is now clear that compounds that have this pharmacology also have anti-inflammatory (Chawla et al. 2001; Cuzzocreaa et al. 2004) and cell cycle effects (Palakurthi et al. 2001) that are clearly not dependent on PPARγ. Moreover, TZDs rapidly activate AMP-kinase in vivo in a process that is not reliant on activation of PPARγ (LeBrasseur et al. 2006). It is not clear whether these effects may be mediated by a recently identified mitochondrial binding site for the TZDs (Colca et al. 2004) or whether the non-PPARγ-mediated pharmacology may participate in the insulin sensitizing effects of these compounds. One way to further define which mechanisms are important for the beneficial pharmacology of these compounds is to directly compare close analogs that vary in their ability to activate PPARγ—driven gene transcription in vitro and in vivo. The current studies address the general effects of two such similar analogs in an effort to find the key elements of the transcriptional regulation that are correlated with insulin sensitization.

## Materials and Methods

### Experimental protocol

Aged-matched male C57 and KKAy mice (8 weeks of age) were obtained from Jackson Laboratories, acclimated to ad libitum water and mouse chow for one week and then dosed with a single oral dose of vehicle (1% sodium methyl carboxycellulose/0.01% Tween 20) or thiazolidinedione suspension daily. Ancillary experiments showed that these conditions and doses produced maximum glucose and insulin reductions in the diabetic KKAy mice for the respective compounds. Following the seventh daily dose, the mice were fasted for 4 hours, anesthetized with isoflurane, blood samples were taken from the orbital sinus and tissue samples were immediately frozen and stored at −80 °C until the RNA could be extracted for RT-PCR and transcriptional profiling. Plasma was immediately separated and analyzed for glucose using an auto-analyzer and insulin by radioimmunoassay (Linco, St. Louis). Total RNA was isolated using Qiagen RNeasy. RNA was amplified for microarray using Ambion Message-AmpII. Pre and post-amplification RNAs were verified for integrity using Agilent Bioanalyzer Nano chip. Liver samples were used primarily for aP2 expression as a biomarker for PPARγ-activation. Adipose tissue RNA was taken as a measure of global transcriptional changes. The animal studies were reviewed and approved by the Pfizer Institutional Animal Care and Use Committee. The animal care and use program is fully accredited by the Association for Assessment and Accreditation of Laboratory Animal Care, International.

### Message and microarray analysis

Real-Time RT-PCR reagents, software and equipment were purchased from Applied Biosystems. Taqman reactions were performed in triplicate using One-Step RT-PCR Master Mix Reagents with the ABI PRISM^®^7900 Sequence Detection System. Gene expression was calculated using the comparative CT method.

Microarray analysis was performed on Agilent Technologies Whole Mouse Genome Microarrays. Amplified RNA was labeled with reagents from the Micromax ASAP RNA labeling kit from Perkin Elmer. Microarray images were acquired using the Agilent Technologies Microarray Scanner. Data was extracted using Imagene 5.6 from Biodiscovery. Data underwent a rigorous QC including background normalization and segmented regression.

### Assays in vitro

The luciferase reporter assay was carried out in HUH7 cells bulk-transfected using Fugene-6 to express a Gal4-PPARγ-LBD construct and a Beta-Gal control plasmid as described (Vosper et al. 2003). Cells were incubated overnight with 6 wells per dilution point. Data are a mean of three independent experiments. Statistical significance was determined by Student’s t-test for PNU-91325 versus Rosiglitazone by comparing activation of luciferase at 1.2, 12, and 33uM. Similar data have been obtained in CHO and HepG2 cells and in a PPARγ SPA assay (not shown).

Binding of 3H-pioglitazone to solubilized rat liver mitochondrial membranes and crosslinking of crude liver mitochondrial membranes were conducted as previously described (Colca et al. 2004).

Activation of AKT was measured as follows: HUH7 cells were plated in 96-well plates at a density of 15K/well and incubated overnight in growth medium to allow attachment. Cells were serum-starved overnight then treated with or without TZDs for four hours and with or without insulin (1nM) for 5 minutes prior to harvest. In some experiments one half of the cells were treated with 100nM wortmannin for 1 hour before TZD treatment to determine the dependence on activation of PI-3 kinase. In other experiments, half of the cells were also treated with the PPARγ antagonist T0070907 (Lee et al. 2002) (TLK) 1-hour before TZD treatment to determine whether the activation of PPARγ was required. Following all treatments, the cells were harvested and lysates were transferred to Meso Scale Discovery’s (MSD) Multi-Spot plates where both total and phosphorylated (Ser473) AKT were measured according to the manufacturer’s directions. MSD’s technology employs a modified sandwich ELISA format that utilizes electrochemiluminescence technology, affording the detection of multiple proteins in a multiplexed format.

## Results

### PNU-91325 and rosiglitazone comparisons in vitro

**Figure 1:**
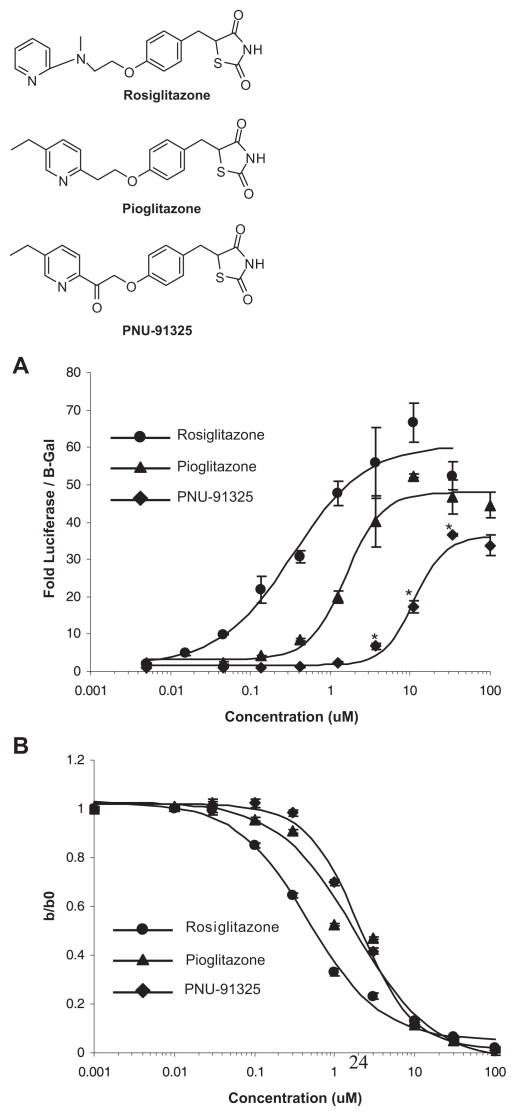
PNU-91325, pioglitazone and rosiglitazone activation of Gal4-PPARγ versus mitochondrial binding. The structures of the thiazolidinediones used in these studies are shown (Top to bottom: rosiglitazone, pioglitazone and PNU-91325). **A.** (upper panel) HUH7 cells expressing Gal4-PPARγ were treated with doses of thiazolidinediones shown on the abscissa and the luciferase response is recorded on the ordinate as discussed in the text. The data are an average and SEM of triplicate experiments with this cell type. *p < 0.05 versus rosiglitazone at the same concentration. Similar data were obtained on other occasions with CHO and HepG2 cells (not shown). **B.** (lower panel) Solubilized liver mitochondrial membranes were incubated with ^3^H-pioglitazone in the combined presence of the concentration of the compounds shown on the abscissa and as discussed in the text. The data are plotted as the ratio of specific counts without competition to those in the presence of the indicated concentration of unlabeled competitor. These data are average of triplicate points and SEM (note: most SEMs fall within the symbols) of a single experiment, but are representative of more than three experiments with liver mitochondrial membranes.

In the process of evaluating possible oxidative metabolites of pioglitazone, a ketone, PNU-91325, was synthesized that is not an actual metabolite of pioglitazone (Tanis et al. 1996). Preliminary studies indicated that this compound had similar pharmacology to pioglitazone (Tanis et al. 1996), but subsequent evaluations demonstrated very weak activation of PPARγ. Published metabolomics studies show this compound may increase mitochondrial fatty acid oxidation in vitro more than pioglitazone and rosiglitazone (Harrigan et al. 2006). Figure 1A shows a direct comparison of the two approved anti-diabetes drugs versus PNU-91325 with respect to activation of Gal4-PPARγ-driven expression of a luciferase reporter in vitro. Compared to rosiglitazone, PNU-91325 is a weaker PPARγ activator with approximately two orders of magnitude reduced affinity, but with similar ability to compete with the mitochondrial pioglitazone binding site (Figure 1B). All of these analogs also competed in a similar fashion for the crosslinking of mitoNEET as described (Colca et al. 2004) (not shown). The structures of the compounds are shown in the inset to Figure 1.

**Figure 2:**
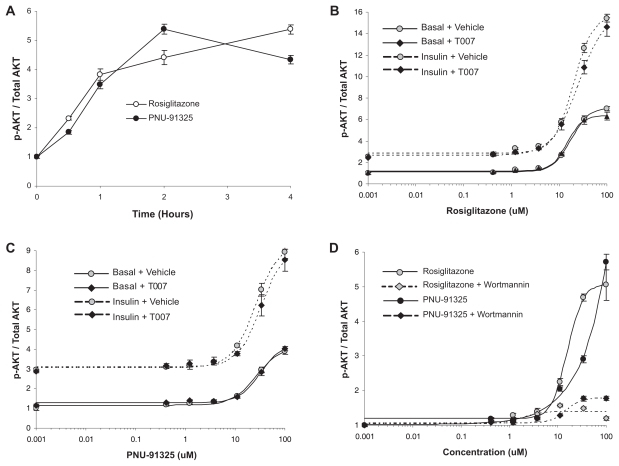
Direct increase in p-AKT in vitro is not dependent on PPARγ activation. HUH7 cells were treated and phosphorylated AKT was measured and expressed as a ratio to total AKT as described in the text. **A.** Shows the time course of increased content of pAKT with rosiglitazone (open symbols) and PNU-91325 (closed symbols). The time of exposure to the TZDs is shown on the abscissa and the ratio of the increase in pAKT/total AKT is shown on the ordinate. Data are mean and SEM of triplicate determinations. **B.** Rosiglitazone activation of AKT is not blocked by a PPARγ antagonist. Wells were pretreated for 1 hour with (solid symbols) or without (open symbols) the PPARγ antagonist T0070907 (10 μM) and then treated with the concentrations of rosiglitazone shown on the abscissa for 4 hours. Half of the wells were also treated with half-maximal insulin (1 nM; dotted lines) 5 minutes before harvesting the cells for measurement of the content of phosphorylated AKT as described in the text. The data are the mean and SEM of triplicate wells from a representative experiment. **C.** PNU-91325 activation of AKT is not blocked by a PPARγ antagonist. The same experimental protocol and data presentation as in Panel B except that the TZD used was PNU-91325. **D.** Activation of AKT by TZDs is blocked by wortmannin. The experimental protocol and data presentation are the same as those described in Panel A except that wortmannin was added 1 hour before rosiglitazone or PNU-91325.

Both PNU-91325 and rosiglitazone acutely activated the level of AKT phosphorylation in HUH7 cells (Fig. 2). This activation occurred in the absence and in the presence of a maximally effective dose of insulin and appeared to be synergistic with insulin (Fig. 2B and 2C). This acute action of both TZDs was not dependent on activation of PPARγ as it was not affected by pre-incubation with 10 μM of the PPARγ inhibitor T0070907 (Vosper et al. 2003) (Fig. 2B and 2C solid symbols versus open symbols). Under these conditions, T0070907 completely prevented the PPARγ response as measured in Figure 1A (not shown). The increased phosphorylation of AKT likely occurred secondary to activation of PI-3 kinase since the effect was largely prevented by the inclusion of the PI-3 kinase inhibitor wortmannin (100 nM, Figure 2D).

### PNU-91325 and rosiglitazone comparisons in vivo; lack of correlation of improved insulin sensitivity with activation of PPARγ

Given the interesting differences between PNU-91325 and rosiglitazone in vitro with respect to activation of PPARγ-driven transcription, a comparison was undertaken in vivo to determine whether changes in gene expression could provide some further insight into the pharmacology of these compounds. In the first comparisons, an evaluation was made into the relative ability of these compounds to activate PPARγ—driven transcription in vivo to establish whether the differences that were observed in vitro would also occur in vivo. A secondary analysis of the tissues from these animals took a more global view to look for correlations and differences in these two TZDs that vary in their ability to activate PPARγ.

**Figure 3:**
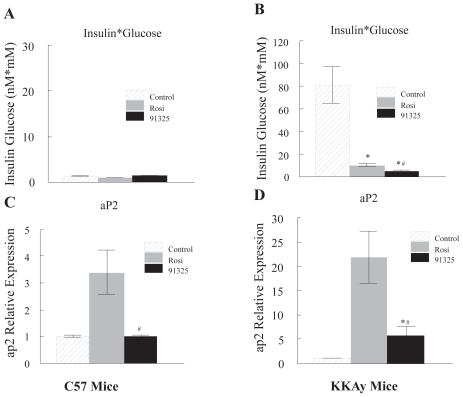
Improvement of insulin sensitivity as compared to activation of the PPARγ biomarker, aP2, produced by PNU-91325 and rosiglitazone. C57 normal mice and diabetic KKAy mice were treated orally with a single maximally effective dose of rosiglitazone (20 mg/kg), PNU-91325 (100 mg/kg) or vehicle for 7 days. On the 8th day and after a 4 hour fast, circulating glucose and insulin levels were measured. Fasting levels of glucose and insulin averaged 22.7 mM and 3.7 nM, respectively for the diabetic KKAy mice and 12 mM and 0.1 nM, respectively for the non-diabetic C57 mice in the control (vehicle) groups. The product of insulin (nM) and glucose (mM) is presented as an index of any changes in insulin sensitivity. This statistic is shown for the normal mice in Panel **A** and for the diabetic KKAy mice in Panel **B** (mean and SEM; n = 8). The relative expression of the PPARγ biomarker aP2 in the liver of C57 mice (mean and SEM; n = 8) is shown in Panel **C** and the relative expression of aP2 in the liver of KKAy mice (mean and SEM; n = 8) is shown in Panel **D**. As expected, there was not a measurable change in insulin sensitivity in non-diabetic mice. Whereas this dose of PNU-91325 produced a similar increase in insulin sensitivity as did rosiglitazone (decrease in glucose*insulin), it produced less increase in the expression of the aP2 transcript. *p < 0.05 versus vehicle. # p < 0.05 versus rosiglitazone.

To determine whether PNU-91325 also produced a reduced activation of PPARγ in vivo, mice were dosed with maximally effective doses of PNU-91325 or rosiglitazone, the most effective PPARγ activator of these analogs, or vehicle for 7 days. As discussed in the Methods section, both compounds were dosed to produce their maximal effects on insulin sensitivity. The effects of these thiazolidinediones on insulin sensitivity are shown in Figure 3A. Improvement in insulin sensitivity is shown by the large decrease in the product of circulating insulin and glucose in the thiazolidinedione-treated diabetic KKAy mice. Since, as expected, both insulin and glucose were decreased by both drugs, the product of the two parameters was used to give a single statistic. Tissues from these animals were used for genome-wide transcriptional profiling (see below) and specific message expression levels were also measured by PCR. The expression of hepatic aP2 was taken as a biomarker for the activation of PPARγ—driven transcription. Rosiglitazone-induced transcription of aP2 in the liver is known to be dependent on the expression of PPARγ; this may reflect differentiation of adipose-like cells within the liver (Matsusue et al. 2003, Schadinger et al. 2005, Spiegelman 1998) and it thus provides a sensitive biomarker for activation of the transcription factor in vivo. As seen in Figure 3B, rosiglitazone (20 mg/kg) produced a three-fold increase in aP2 message in liver from normal C57 mice while PNU-91325 produced no change even at a five-fold higher dose. In diabetic KKAy mice which have an increased expression of PPARγ in the liver, the same dose of rosiglitazone produced a more than 20-fold increase in the expression of aP2 while PNU-91325, although yielding a slightly greater increase in insulin sensitivity (Fig. 3A), caused only 25% of the increase in aP2 message observed with rosiglitazone (Fig. 3D). Thus, the relative activation of the PPARγ target biomarker aP2 by maximal doses of the two compounds in vivo reflected the differences in PPARγ—driven transcription in vitro and did not predict the degree of lowering of plasma glucose and insulin.

### Increased expression of messages for mitochondrial proteins correlates with improved insulin sensitivity

**Figure 4:**
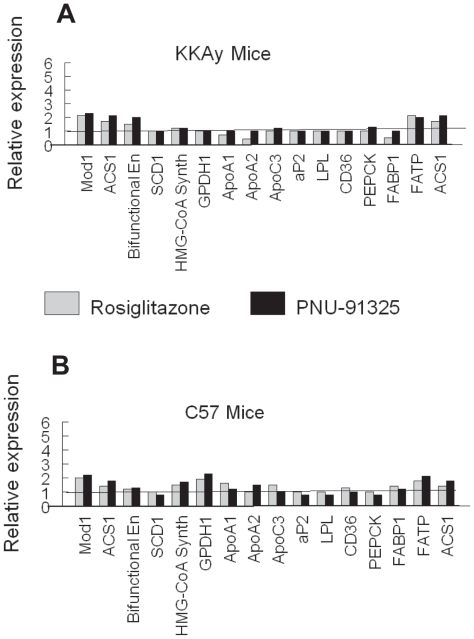
Comparison of regulation of PPRE-regulated genes in adipose tissue from normal and diabetic mice. Data for genes containing PPRE (selected as discussed in the text) were taken from the transcriptional profiling of epididymal fat pads of KKAy (**A**) and C57 mice (**B**) shown in Figure 4. The respective transcript levels were compared for the gene shown on the abscissa in the samples from rosiglitazone-treated mice (gray) and PNU-91325-treated mice (black) versus vehicle (taken as 1). Data are from all 8 mice of each group. Malic enzyme, supernatant (Mod1), [**NM_008615**]; Acyl-CoA synthetase long-chain family member 1 (Acsl1), [NM_007981]; Enoyl-Coenzyme A, hydratase/3-hydroxyacyl Coenzyme A dehydrogenase (Ehhadh), [**NM_023737**]; Stearoyl-Coenzyme A desaturase 1 [**7530417E14**]; 3-hydroxy-3-methylglutaryl-coenzyme A synthase 1. [**NM_145942**]; Glycerol phosphate dehydrogenase 1, **[0610008N20**]; Apolipoprotein A-I (Apoa1), [**NM_009692**]; Apolipoprotein A-II (Apoa2), [**NM_013474**]; Apolipoprotein C-III (Apoc3), [**NM_023114**]; Fatty acid binding protein 4, adipocyte (Fabp4), [**NM_024406**]; Lipoprotein lipase (Lpl), [**NM_008509**]; CD36 antigen (Cd36), [**NM_007643**]; Phosphoenolpyruvate carboxykinase 1, cytosolic (Pck1), [**NM_011044**]; Fatty acid binding protein 1, liver (Fabp1), [**NM_017399**]; Solute carrier family 27 (fatty acid transporter), member 1 [**NM_011977**] (FATP); Acyl-CoA synthetase long-chain family member 1 (Acsl1), [**NM_ 007981**].

To gain further information on the global effects of the two thiazolidinediones to increase PPARγ—driven gene transcription, an evaluation of was made of the relative expression of 16 genes with established PPAR response elements (Desvergne and Wahli, 1999; Juge-Aubry et al. 1997) in adipose tissue, the tissue with the highest expression of PPARγ (Spiegelman, 1998). As seen in Figure 4, there was not a consistent increase in the expression of these 16 genes in adipose tissue from either normal or diabetic mice following treatment with either compound. Thus, there was not a correlation with antidiabetic activity with the increased expression of transcripts known to be activated by PPARγ with either of the TZDs in the metabolically sensitive epidydimal adipose tissue.

**Figure 5:**
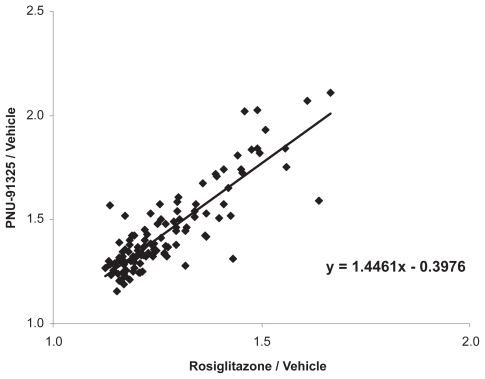
Comparison of the effects of PNU-91325 and rosiglitazone treatment on transcripts for mitochondrial proteins. Data were extracted from the transcriptional profile of >300 mitochondrial proteins from the array data from the mice shown in Figure 3 and 4 and relative increase with each thiazolidinedione versus vehicle were plotted. For each of the transcripts, the relative increase produced by PNU-91325 versus vehicle is plotted on the ordinate as compared to the relative increase in the same transcript produced by rosiglitazone on the abscissa. On average, the effect of PNU-91325 was 1.4 times that of rosiglitazone.

**Figure 6:**
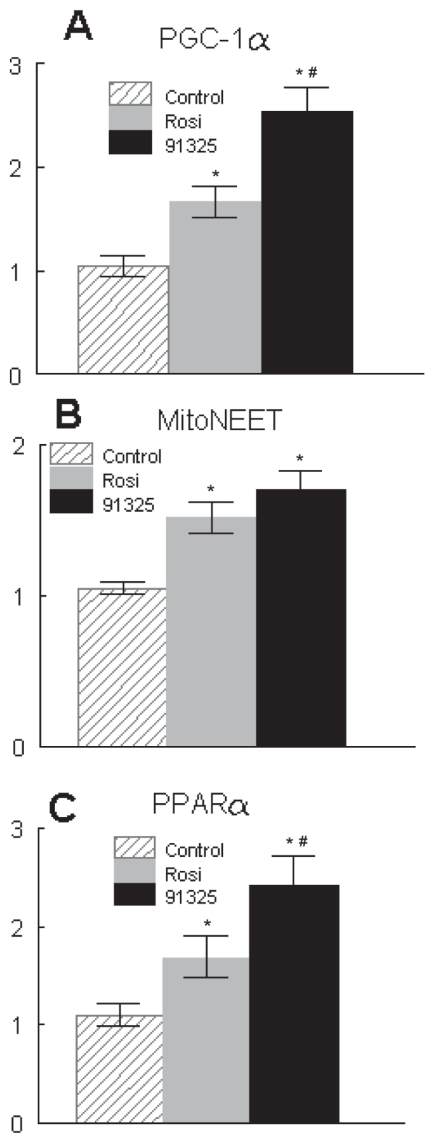
Effects of PNU-91325 and rosiglitazone on expression of PGC1α, mitoNEET and PPARα. RNA was isolated from the epididymal fat pads of diabetic KKAy mice treated with vehicle (lined), rosiglitazone (gray) or PNU-91325 (black) bars. The expression of transcripts relative to vehicle was determined for PGC1α (**A**), mitoNEET (**B**) and PPARα (**C**) (data are mean and SEM; n = 8). *p < 0.05 versus vehicle. # p < 0.05 versus rosiglitazone.

To take an unbiased look for transcripts that might be coordinately regulated by these two TZDs, global transcriptional changes produced by both compounds were evaluated by transcriptional profiling. A perusal of all of these data clearly demonstrated a coordinated increase in transcripts of proteins involved in mitochondrial structure and function. Detailed evaluation of the transcriptional profiles of adipose tissue taken from these mice indicated that there was a general increase in mRNA for over 300 genes coding for mitochondrial proteins. Moreover, the increased insulin sensitivity produced by dosing with PNU-91325 under these conditions correlated with the general increase in these transcripts produced by PNU-91325 relative to rosiglitazone (Fig. 5). That is, there was a small but consistent increase in the effect of PNU-91325 over that of rosiglitazone (1.4-fold), and the increase was similar in magnitude to the increase seen in overall insulin sensitivity under these conditions (2.1-fold, Fig. 3A). These transcriptional profiling results were confirmed by quantitative RT-PCR. Examples of the relative differences measured by RT-PCR are shown for PGC1α, the master regulator of mitochondrial biogenesis (Puigserver and Spiegelman, 2003), and mitoNEET, a putative direct mitochondrial target for the thiazolidinediones (Colca et al. 2004) (Fig. 6A and B). Interestingly, PNU-91325 also produced a greater induction of PPARα (Fig. 6C) which would be expected to drive an increase in the oxidation of free fatty acids and contribute to the decrease in circulating lipids that is seen with this class of compounds (Owens, 2002).

Review of the entire data set (not included here) demonstrates that there are numerous compound-specific effects on relative gene expression, but the common changes that correlate with the ability to improve insulin sensitivity (i.e. lower circulating glucose and insulin concentrations) involve the increased expression of mitochondrial proteins and increased capability to carry out mitochondrial β—oxidation of fatty acids. These actions could be driven by a direct effect to increase mitochondrial biogenesis secondary to activation of AMP-kinase (LeBrasseur et al. 2006; Brunmair et al. 2001). Thus, the key finding of this evaluation is that the insulin sensitizing effects of two structurally similar TZDs correlates not with the activation of the transcription factor PPARγ, but with a global pattern of increased expression of transcripts encoding mitochondrial function.

## Discussion

These studies compare the effects of two structurally similar thiazolidinedione insulin sensitizing agents that vary in their ability to activate the nuclear receptor PPARγ both in vitro and in vivo. The data do not support the simple model that the magnitude of activation of PPARγ regulated gene transcription is the sole basis for the pharmacology of these compounds. Rather, the data are consistent with an alternate hypothesis that non-PPARγ-dependent actions may contribute to the insulin sensitizing pharmacology.

As reviewed in the Introduction, there are a variety of acute actions of the TZDs that have been shown to be independent of activation of PPARγ. To this list we add the ability to activate PI-3 kinase-dependent phosphorylation of AKT. This activation occurred in vitro with or without the addition of insulin and was not affected by blockade of PPARγ—driven transcription (Fig. 2). AKT lies at the center of multiple biochemical control mechanisms that affect hormone and cytokine action and cell survival and this could play an important role in some of the pleiotropic effects of these compounds (Cantley, 2002; Fayard et al. 2005; Ushio-Fukai et al. 1999). It is unknown to what extent this and other acute effects of the TZDs may mediate the overall pharmacology of these compounds, especially those aspects of the pharmacology which are manifested over many days of treatment. It is important to note, however, that many acute effects of the compounds have been reported in the literature (Brunmair et al. 2001; Brunmair et al. 2004; LeBrasseur et al. 2006) that include impact on mitochondrial function. We also confirmed that these TZDs can alter the amount of reactive oxygen and ATP produced by isolated mitochondria (not shown). In support of the hypothesis that acute effects contribute to insulin-sensitizing pharmacology, Lee and Olefsky demonstrated that within 15 minutes there is a statistically significant improvement in insulin-mediated glucose uptake produced by infused troglitazone in intact rats (Lee and Olefsky, 1995). One possibility may be that while there are acute effects of the compounds on molecular processes that begin to improve insulin sensitivity, the full pharmacology depends on longer term gene expression and modification of cellular and mitochondrial function (see below).

It is interesting that the insulin sensitizing pharmacology produced by these two analogs was closely tied to a pattern of gene regulation that suggests a pressure to increase mitochondrial biogenesis. Recent data have confirmed that whereas mitochondrial numbers are decreased in diabetes and insulin resistant states, thiazolidinedione treatment can result in *increased* mitochondrial biogenesis in man (Bogacka et al. 2005). The mechanism for how this may occur remains to be defined and may involve processes that are both dependent and independent of PPARγ. The recent identification of PGC1α as a potentially direct PPARγ target gene suggests a receptor-mediated increase in PGC1α expression could be involved as an initial step leading to increased mitochondrial biogenesis (Hondares et al. 2006). However, as discussed above, there is also evidence for direct mitochondrial effects of the TZDs (Brunmair et al. 2001; Brunmair et al. 2004; Crosby et al. 2005; Feinstein et al. 2005). Furthermore, new data suggest that direct activation of AMP-kinase, known to occur with TZD treatment, is capable of increasing the expression of both PPARα and PGC-1 (Lee et al. 2006), both of which are key regulators of fatty acid oxidation. It is possible that TZDs may affect redox signaling that results in the activation of redox-sensitive enzymatic machinery such as AKT, PTEN, AMP-kinase, etc. Interestingly, the mitochondrial protein that may participate in TZD signaling in mitochondria (Colca et al. 2004) has been suggested to have a redox sensing and/or transducing function (Colca, 2006). Recent evidence suggests that this protein, also called mitoNEET, is an iron-containing protein that is in the outer mitochondrial membrane and plays a key role in the regulation of oxidative metabolism (Wiley et al. 2007).

The current data suggest that increased transcription of mitochondrial proteins is a major feature of two compounds that differ widely in their ability to activate PPARγ but which share similar pharmacology. Thus, key pharmacology of these compounds may not require direct activation of the nuclear transcription factor PPARγ. Since it is now clear that thiazolidinedione-induced edema, the main dose-limiting side effect of these compounds in patients, is likely secondary to activation of PPARγ (Guan et al. 2005; Zhang et al. 2005), these results suggest a potential way to generate improved therapeutics with this mode of action.
